# A Review of Application of Deep Learning in Endoscopic Image Processing

**DOI:** 10.3390/jimaging10110275

**Published:** 2024-11-01

**Authors:** Zihan Nie, Muhao Xu, Zhiyong Wang, Xiaoqi Lu, Weiye Song

**Affiliations:** 1 School of Mechanical Engineering, Shandong University, Jinan 250061, China; 202414371@mail.sdu.edu.cn (Z.N.); ujnmhxu@hotmail.com (M.X.); wzy2021@mail.sdu.edu.cn (Z.W.); 17604135762@163.com (X.L.); 2 Key Laboratory of High Efficiency and Clean Mechanical Manufacture of Ministry of Education, Shandong University, Jinan 250061, China

**Keywords:** deep learning, endoscopy, image analysis, convolutional neural networks (CNNs)

## Abstract

Deep learning, particularly convolutional neural networks (CNNs), has revolutionized endoscopic image processing, significantly enhancing the efficiency and accuracy of disease diagnosis through its exceptional ability to extract features and classify complex patterns. This technology automates medical image analysis, alleviating the workload of physicians and enabling a more focused and personalized approach to patient care. However, despite these remarkable achievements, there are still opportunities to further optimize deep learning models for endoscopic image analysis, including addressing limitations such as the requirement for large annotated datasets and the challenge of achieving higher diagnostic precision, particularly for rare or subtle pathologies. This review comprehensively examines the profound impact of deep learning on endoscopic image processing, highlighting its current strengths and limitations. It also explores potential future directions for research and development, outlining strategies to overcome existing challenges and facilitate the integration of deep learning into clinical practice. Ultimately, the goal is to contribute to the ongoing advancement of medical imaging technologies, leading to more accurate, personalized, and optimized medical care for patients.

## 1. Introduction

With the rapid development of technology, deep learning (DL) has demonstrated its transformative potential in various fields. From intelligent transportation systems (ITSs) to environmental monitoring, deep learning has not only improved the efficiency of traditional processing methods but also promoted the development of automation and intelligence [1,2]. These advances not only bring new opportunities to specific fields but also lay the foundation for the development of future cross-domain intelligent systems.

In modern medicine, endoscopy has become an increasingly vital tool, significantly enhancing the precision and efficiency of early disease diagnosis and treatment through direct visualization of internal organs [3]. Its applications in fields such as the digestive, respiratory, and urinary systems have made it a crucial method for both diagnosis and treatment. Endoscopic technology not only enables real-time observation of internal organs but also allows for the evaluation of lesions by imaging biological tissues, providing more accurate diagnostic information [4]. With the rapid advancements in endoscopy, the amount of data generated is also increasing, making efficient processing and analysis of this data essential for improving diagnostic accuracy and treatment outcomes. However, traditional image processing methods often suffer from slow speeds and limited accuracy, reducing their effectiveness in handling complex medical data [5].

To address these challenges, researchers have increasingly turned to deep learning technologies for endoscopic image analysis, significantly improving both efficiency and accuracy. Convolutional neural networks (CNNs), in particular, have brought revolutionary improvements to image analysis by mimicking the brain’s processing mechanisms. CNNs automatically learn and extract key features from images, enabling efficient and high-precision analysis [6]. This technology has enhanced capabilities in image segmentation, classification, and anomaly detection, reducing the workload for physicians. Computer-aided detection (CAD) and computer-aided diagnosis (CADx) can find or localize abnormal or suspicious areas in structural images, assisting doctors in making faster and more accurate decisions [7].

The quality of images is crucial to the success of deep learning-based analysis and diagnosis. In recent years, several studies have focused on enhancing the quality of endoscopic images using deep learning techniques such as image enhancement, noise removal, and resolution improvement. For instance, certain algorithms are able to create high-quality synthetic medical images or generate missing information, thereby improving diagnostic accuracy [8]. This not only enhances the quality of endoscopic examinations but also reduces the need for repeated procedures, lowering medical costs.

The advances in deep learning technology have also paved the way for personalized medicine. By utilizing deep learning models, researchers can develop more accurate diagnostic tools that provide tailored treatment recommendations based on a patient’s specific condition. These innovations not only improve treatment outcomes but also optimize resource use [9]. As artificial intelligence continues to evolve, future endoscopic examinations are expected to become increasingly intelligent, not only through enhanced image analysis but also via data integration and comprehensive analysis, allowing for personalized medical services, health risk predictions, and tailored preventive measures [9,10,11].

In this paper, we review the application of deep learning technology in endoscopic image analysis. Since deep learning has made significant breakthroughs in image recognition, it has been widely adopted in medical imaging, particularly in feature extraction, disease diagnosis, and image enhancement for endoscopic images. We explore in detail the central role of CNNs in improving disease detection accuracy and processing efficiency, as well as how deep learning reduces physicians’ workload through automated analysis, improving the detection and classification of lesions. Additionally, this paper reviews recent advances in deep learning for image quality enhancement, dynamic tracking, and three-dimensional reconstruction, discussing how these technologies can support clinical diagnosis and treatment. Through this analysis, we aim to demonstrate the potential of deep learning to further advance endoscopic technology and provide insights for future research directions.

## 2. Related Surveys

Several review papers delve into the intersection of artificial intelligence (AI), particularly deep learning, with gastrointestinal endoscopy and broader medical image analysis. Each provides valuable insights, although with differing scopes and focuses.

Shen et al. [7] provide a foundational overview of deep learning in medical imaging, including architectures such as CNNs and generative models. Alagappan et al. [10] adopt a forward-looking perspective, predicting the transformative impact of AI on gastrointestinal endoscopy. Choi et al. [8] examine the role of convolutional CNNs in endoscopic imaging, particularly for polyp detection and classification. Their review emphasizes the technical development and challenges related to CNN applications in diagnostic imaging. Pannala et al. [9] emphasize the real-time potential of AI, particularly through computer-aided detection (CADe) systems that aim to improve adenoma detection rates. Zhuang et al. [12] offer a focused examination of deep learning applications within digestive system imaging, analyzing recent advances.

Across these reviews, several common limitations emerge. None comprehensively tackle the full spectrum of challenges associated with deep learning integration in clinical workflows. Additionally, key technical aspects like super-resolution, noise reduction, and cross-modality integration—essential for achieving higher diagnostic accuracy—are either mentioned briefly or overlooked. Many reviews also focus narrowly, either on future potentials or current applications, without sufficiently bridging the gap between recent research advancements and the practical strategies needed for clinical adoption. This leaves a need for more holistic perspectives that encompass both technical progress and real-world challenges. Table 1 shows the comparison of the reviews.

## 3. Literature Review

### 3.1. Endoscopic Imaging Technology

Endoscopic imaging technology is a core tool in modern medical diagnosis, enabling doctors to directly visualize internal structures of the human body through minimally invasive or non-invasive methods. These technologies are crucial for early disease diagnosis, treatment monitoring, and surgical navigation. Below are five common endoscopic imaging techniques.

#### 3.1.1. White Light Endoscope (WLE)

White light endoscopes (WLEs) are the most widely used endoscopes, utilizing traditional white light illumination to provide high-definition views of the surface structures within body cavities such as the digestive and respiratory tracts. The fundamental principles of white light endoscopy were first established through early studies, including those related to digestive tract diseases [13]. In recent years, WLE technology has continued to evolve, with advancements such as high-resolution endoscopes and video endoscopy systems, which enhance image resolution and detail. For example, modern high-definition WLEs can more accurately detect small lesions, such as intestinal metaplasia [14]. Additionally, combining WLEs with computer-aided diagnostic (CAD) systems that implement discriminant analysis of pit patterns, using quantitative features from training images, has improved the accuracy and efficiency of lesion detection [15].

#### 3.1.2. Intravascular Ultrasound (IVUS)

Intravascular ultrasound (IVUS) involves inserting an ultrasound probe into blood vessels to obtain images of the vessel walls and surrounding tissues, and is widely used to evaluate atherosclerosis and guide endovascular procedures. This technology has been recognized by the Society for Cardiac Angiography and Interventions [16]. With ongoing technological advancements, IVUS now offers improved image quality and expanded applications. High-frequency and three-dimensional IVUS enhance the resolution and accuracy of plaque analysis. For instance, high-frequency IVUS provides finer details of vascular structures, helping doctors better assess plaque composition [17]. Moreover, combining IVUS with other imaging modalities, such as optical coherence tomography (OCT), has further improved the evaluation of spontaneous coronary artery dissection and characterization of atherosclerotic plaques [18,19].

#### 3.1.3. Endoscopic Ultrasound (EUS)

Endoscopic ultrasound (EUS) combines endoscopy and ultrasound to obtain high-resolution images from within the body. It is particularly useful for diagnosing diseases of the gastrointestinal tract and adjacent structures, such as pancreatitis or pancreatic cancer [20]. Recent advancements in EUS include elastography, where a transducer sends a shear wave through the pancreas and generates an elastogram by calculating the velocity of the wave passing through soft tissues [21].

#### 3.1.4. Optical Coherence Tomography (OCT)

OCT uses light waves for cellular-level imaging, commonly applied in ophthalmology and cardiovascular diagnostics. OCT can generate microscopic three-dimensional images of tissue structures, aiding doctors in assessing pathological changes. First proposed by Huang et al. in 1991 [22], OCT has recently been adapted for endoscopic use. For example, wide-field OCT in endoscopy now provides high-resolution images over larger fields of view through improvements in optical design and data processing [23]. The latest advancements, such as optical coherence tomography angiography (OCT-A), show great potential for endoscopic applications [24].

#### 3.1.5. Narrow-Band Imaging Endoscopy (NBI)

Narrow-band imaging (NBI) uses filtering technology to enhance endoscopic imaging by isolating specific blue and green wavelengths. This technology produces images with unique color distributions, aiding in the visualization of tissue structures [25]. NBI has made significant strides, especially in the early detection of gastrointestinal cancers [26].

Together, these endoscopic imaging technologies offer detailed internal views for various clinical scenarios, significantly improving the efficiency and accuracy of disease diagnosis and treatment. As technology continues to advance, these imaging techniques will play an even greater role in medical diagnostics.

### 3.2. Deep Learning Models

In medical image analysis, deep learning technologies, especially convolutional neural networks (CNNs)—have demonstrated tremendous potential. Deep learning mimics the human brain’s ability to process complex data, such as images and sounds [27,28]. CNNs, with their multi-layer convolutional structures, automatically extract features from raw images. Convolutional layers use learnable filters (kernels) to capture local image features, while pooling layers reduce the size of feature maps to decrease computational complexity and retain essential information [29]. Non-linear activation functions (e.g., ReLU) further enhance the model’s ability to recognize complex patterns [27].

In endoscopic image analysis, CNNs’ automatic feature learning greatly improves the efficiency and accuracy of image processing. Studies have shown that CNNs can effectively perform tasks such as polyp detection, lesion classification, and region recognition. For example, a hybrid model combining CNNs with Swin Transformers was able to automatically segment polyps by learning from annotated endoscopic images, demonstrating high efficiency and accuracy when analyzing new images [30].

Additionally, deep learning has proven valuable in enhancing image quality, an important factor in improving the diagnostic value of endoscopic images. For example, using ImageNet-based technologies, researchers have improved image contrast and resolution, leading to better visualization of details and abnormalities [29]. EndoL2H, a model combining conditional adversarial networks with spatial attention, has been developed to achieve super-resolution endoscopic imaging, further enhancing diagnostic accuracy [31]. Deep learning-based denoising techniques have also shown excellent performance in reducing noise, advancing endoscopic image processing technology [32].

As CNN architectures and learning algorithms continue to be optimized, the accuracy and efficiency of endoscopic image analysis improve. For instance, deep residual networks (ResNet) have enhanced the training stability and performance of CNNs, yielding promising results in practical applications [33]. Multi-scale feature fusion and attention mechanisms have also contributed to better capturing subtle changes in endoscopic images [34].

Transfer learning techniques are another area of progress. By employing nucleus-centered image block sampling strategies and combining the classification results of multiple image blocks, researchers have improved robustness and accuracy in endoscopic image classification [35]. CNNs, therefore, play a pivotal role in improving image quality, detecting small lesions, and assisting doctors in making more accurate diagnoses [36,37].

In summary, the application of deep learning in endoscopic image analysis not only increases the automation level of image processing but also accelerates advancements in medical diagnostic technology. Continuous development and optimization of these technologies offer more accurate diagnostic support to clinicians, aiding early disease detection, treatment planning, and treatment monitoring [36].

### 3.3. Application of Deep Learning in Endoscopic Image Processing

To help readers quickly grasp the latest developments in deep learning in endoscopic image processing, Table 2 is an overview table showing the various application areas and their most popular models.

#### 3.3.1. Image Segmentation

Image segmentation is one of the core applications of deep learning in medical image analysis. Identifying and extracting specific regions or structures in images significantly improves the accuracy and efficiency of medical diagnosis. Deep learning models, especially CNNs, have been widely used in various medical image segmentation tasks, such as retinal layer segmentation, lung nodule detection, and cardiac image analysis. For example, a retinal segmentation method demonstrates the practicality of the model in processing actual medical images, especially in clinical environments where fast feedback is required, and efficiency improvement is crucial [50]. Another study proposed a CNN model optimized by multi-granularity visual features, which has made significant progress in improving the accuracy and speed of retinal layer segmentation [51]. The fusion of multi-granular features not only enhances the recognition ability of the model but also provides new perspectives for processing information at different levels. These medical image segmentation techniques provide valuable ideas and references for the segmentation of endoscopic images, laying the foundation for future work in this area.

In the field of gastrointestinal endoscopic image segmentation, deep learning technology has made substantial progress. In a review of the detection of precancerous lesions in upper gastrointestinal endoscopy, CNNs and transfer learning techniques were shown to be highly effective, with different models being evaluated for their efficacy in diagnosing precancerous lesions, thus highlighting the potential of deep learning in early cancer diagnosis [38]. This evaluation provides practical guidance for model selection in clinical practice.

The DeepPoly model uses a DoubleU-Net architecture which stacked one U-net network on another U-net network to perform polyp segmentation and classification. This approach efficiently utilizes semantic information to optimize the segmentation of different objects in medical images, achieving a mean dice-coefficient of 0.834 and 0.956 in segmentation for the Endotech challenge and Kvasir-SEG dataset [39]. This method, trained on a large-scale colonoscopy image dataset, demonstrates that the performance of deep learning models is closely tied to dataset size. Transfer learning alleviates the problem of insufficient data while maintaining high-precision detection effects. Using step-by-step transfer learning and five independent endoscopy datasets, a computer-assisted system was used to characterize tissues in NBI zoom images in Barrett’s esophagus. The results overlapped with the expert ground truth, and the soft and sweet spot delineation scores reached 98% [52]. This result shows that transfer learning can maintain the detection accuracy of the model under limited data conditions, providing an effective solution for actual clinical applications.

**Figure 1:**
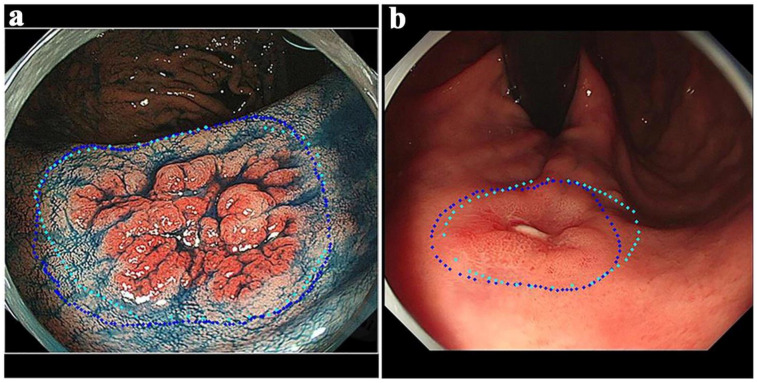
Representative images of the resection extent. (**a**) ENDOANGEL delineates the resection extent in the CE images. (**b**) ENDOANGEL delineates the resection extent in the WLE images. The dark dotted line is the predicted resection margin predicted. The light dotted line is the resection margin delineated by the expert (all images taken from [53]).

Another example is a deep learning method based on UNet++ and VGG-16, used for accurately delineating the resection margins of early gastric cancer. By improving the accuracy testing method, which reconstructs the results on the endoscopic image and analyzes the distances between point pairs, this method significantly improved the delineation accuracy of the resection margin [53]. This progress reflects the importance of adaptability and flexibility in deep learning models, especially as application scenarios in medical imaging expand. The representative images of the resection extent are shown in Figure 1.

In addition, a semantic segmentation model based on SegNet, which is known for its high internal efficiency, has been applied for the detection of bleeding areas in capsule endoscopy images. Unlike traditional networks that output images in RGB, this model produces multiple channels for enhanced image interpretation. The training images were labeled with three classes—bleeding, non-bleeding, and background—and the model significantly improved the detection accuracy of bleeding areas [40], demonstrating the potential of deep learning in processing complex medical images.

In respiratory endoscopic image analysis, a fully automatic vocal fold segmentation system based on deep learning maps three-channel RGB images into distance maps for training. This system analyzes vocal cord motion behaviors captured using flexible endoscopes with low-speed capability, segmenting the vocal cords and glottis areas in transnasal flexible laryngoscopy videos. The segmentation accuracy of this method reaches 0.9469, which is higher than 0.7358 of the encoder–decoder method [54]. This study reveals the significant potential of deep learning in deepening our understanding of complex physiological structures. Another study focused on the segmentation of airways and obstructive factors in endoscopic imaging, providing valuable insights for diagnosing and treating obstructive sleep apnea (OSA) [55]. However, the limited number of images in the test set may affect the generalizability of the model.

Though deep learning can perform image segmentation for various human endoscopy images, the occlusion of human tissues and organs by medical devices will affect the analysis and judgment when performing image analysis. For accurate segmentation of surgical instruments, DRR-Net, a dense-connected residual recurrent convolutional network, has been shown to perform well in instrument recognition within complex backgrounds [56]. This network adds Recurrent Neural Network (RNN) blocks and Adaptive Dilated Recurrent Convolutional Block (ADRCB) blocks to the traditional U-Net network backbone, allowing for time series analysis of video data. By combining data from previous frames and learning long-range context information, DRR-Net reflects the potential of deep learning to improve surgical safety and precision in real-world surgical scenarios. This feature suggests that the model can be integrated with a robotic system in the future to achieve fully autonomous surgical operations. Moreover, complex medical imaging environments can also pose challenges for image analysis. EndoUDA is a modality-independent deep learning segmentation method that leverages adaptive techniques and a joint loss function to improve generalization across different target domains, showing strong potential for wide-ranging applications in various endoscopic image modalities [57]. This research highlights deep learning’s ability to find effective solutions across diverse medical imaging environments.

Artifact detection and segmentation are important challenges in deep learning applications. Artifacts are fundamental yet unavoidable problems in endoscopy, often complicating the detection of tissue abnormalities. Therefore, research on accelerating the identification of these categories and restoring frames is crucial [58]. An improved Cascade R-CNN model, combined with a feature pyramid network (FPN), has been employed for multi-class artifact detection. This combination improves the trade-off between mean average precision (mAP) and intersection over union (IoU), effectively enhancing artifact recognition and segmentation accuracy [59]. These studies highlight the importance of developing specialized techniques to handle artifacts, which in turn enhances the overall image quality and diagnostic level in endoscopy.

**Figure 2:**
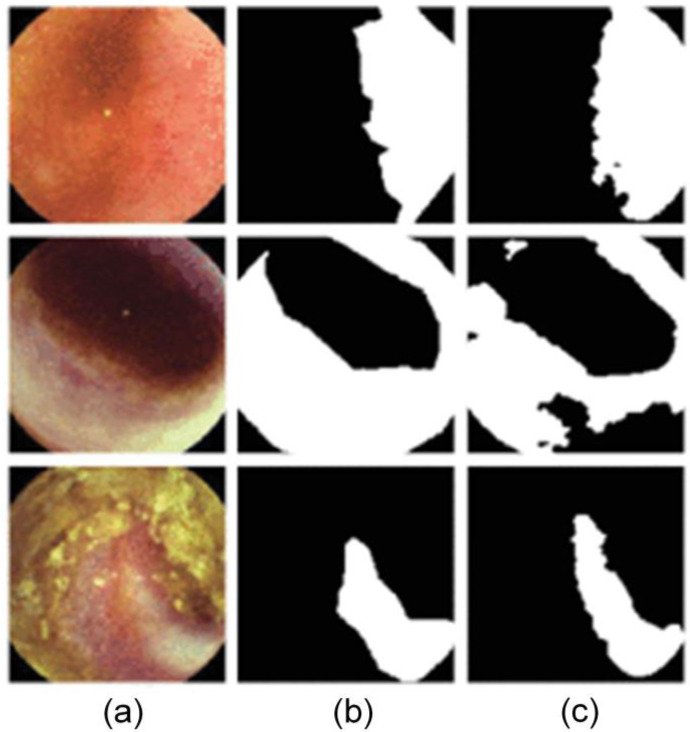
Example of DPENet segmentation results. White area: lesion. Black area: background. (**a**) The data collected by endoscopes (**b**) ground truth, (**c**) segmentation results of DSI-Net (all images taken from [61]).

While expanding the types of segmentable images, it is also crucial to improve the integration of tasks. Multi-task deep learning models have also gained widespread use. A multi-task model that combines classification, image retrieval, and segmentation functions utilizes a mutual attention module to capture more diverse features during the segmentation task, significantly improving the accuracy and efficiency of endoscopic image analysis [60]. This type of multi-task learning approach not only enhances the overall performance of the model but also reduces the time and data required for training. Another model, DSI-Net, combines classification and segmentation tasks, significantly improving the multi-task processing capability of endoscopic images through deep collaborative interactive networks and attention mechanisms [61]. An example of DPENet segmentation results is shown in Figure 2.

In summary, these research findings demonstrate that the application of deep learning in endoscopic image segmentation not only improves the accuracy of lesion detection and segmentation but also drives the overall progress of endoscopic technology. The continuous development and application of these technologies provide robust support for clinical diagnosis and treatment, underlining the crucial role of deep learning in medical image analysis.

#### 3.3.2. Image Classification

The application of deep learning techniques in endoscopic image classification has made significant progress. These techniques not only enhance the efficiency of diagnosis but also expand the scope of application for endoscopic technology. In the field of deep learning-based endoscopic image classification, CNNs have shown great potential [62].

For example, in the classification of gastroesophageal reflux disease (GERD), researchers designed a CNN architecture with high generalization ability, using a pre-trained model and applying dynamic data augmentation to the training set. This model classified GERD according to the Los Angeles classification (LA grade) system, achieving an accuracy of 89.3% [41]. The high generalization ability of the model was enhanced by the use of a data augmentation technique, a two-stage no-freezing fine-tuning policy, and an early stopping criterion technique [63].

**Figure 3:**
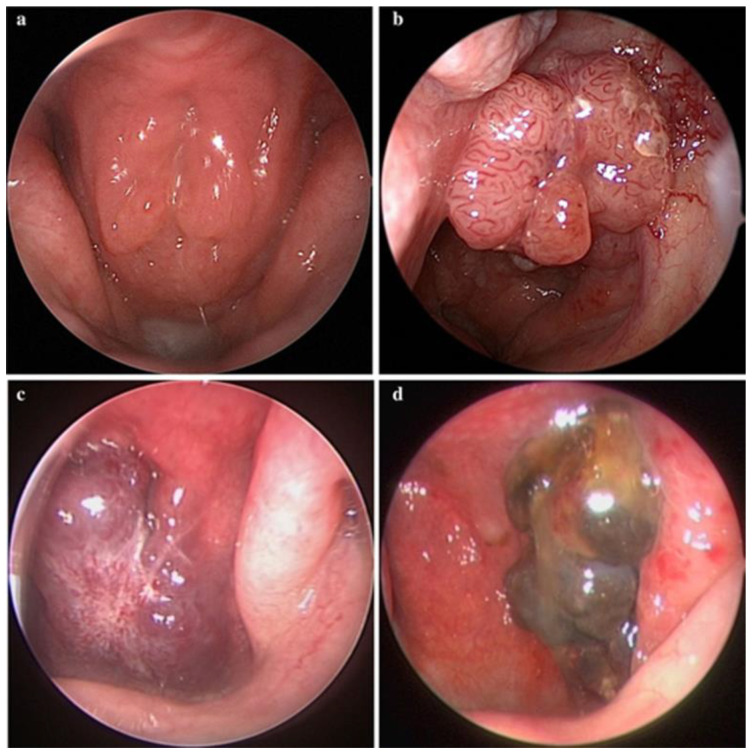
Representative images of nasopharyngeal masses. (**a**) Normal (adenoids hyperplasia); (**b**) nasopharyngeal carcinoma; (**c**) fibroangioma; (**d**) malignant melanoma (all images taken from [42]).

Accurate detection of abnormalities is essential for effective diagnosis and treatment in medical imaging. A study utilized deep learning models consisting of a fully convolutional network based on the inception architecture to detect nasopharyngeal malignancies and verified the effectiveness and reliability of the model in real clinical settings, achieving an accuracy of 88.7% on the test set—7.5% higher than the expert in the prospective comparison phase [42]. Representative images of nasopharyngeal masses of different types are shown in Figure 3. The success of this study highlights the maturity of deep learning technology in the analysis of various endoscopic images and suggests that it may become a routine diagnostic tool in the future.

Another study developed a guided attention deep network based on ResNet-50 which combined a lightweight attention module and multi-scale feature extractor with U-Net. By extracting image features at different scales, the model significantly improved the recognition ability for early gastric cancer, achieving a classification accuracy of 92.2% [43]. However, this method’s data processing range remains somewhat limited, and its recognition ability may be constrained when dealing with data from diverse regions. Ensemble learning can deal with this problem of poor robustness, another study found that the performance of pathological site classification could be enhanced through ensemble learning, a method using multiple network architectures and CNNs of different depths to effectively extract image texture features from input images. This technique not only compensates for the biases of individual models but also reduces error rates by integrating the prediction results of multiple models [64]. Building on this concept, Ghosh et al. (2023) further demonstrated that ensemble learning can successfully classify features that were incorrectly learned by individual base learners, improving accuracy to 95% [65].

Meanwhile, by adding a global spatial feature aggregation block to the ResNet-50 backbone, researchers were able to accurately aggregate spatial features, demonstrating excellent classification performance [66]. This improvement is particularly important for the positioning and analysis of complex tissues. In the future, it can be integrated with the smart capsule endoscopy system to achieve non-invasive detection and monitoring. A recent study by Mukhtorov et al. (2023) improved the transparency of endoscopic image classification through the development of interpretable deep learning models. This innovation is particularly important for enhancing clinicians’ trust in AI-powered diagnostic tools [67].

In summary, the combination of different models and methods not only improves classification accuracy but also broadens the potential for clinical application in endoscopic diagnosis.

#### 3.3.3. Image Enhancement

Image enhancement is a crucial application of deep learning in medical image processing, particularly in endoscopic image analysis. By improving the quality of images, enhancement techniques enable doctors to identify lesions more accurately, thereby increasing diagnostic accuracy and efficiency. In recent years, significant progress has been made in this field through deep learning methods.

**Figure 4:**
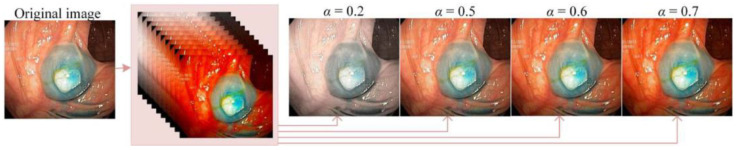
The visual effect of different α (parameter variable in adaptive adjustment function for changing saturation) values on image (all images taken from [44]).

For colonoscopy images, the endoscopic image enhancement network (EIEN) based on Retinex theory has demonstrated successful illumination correction using a self-attention-guided multi-scale pyramid network. This approach stretches the green and blue channels in the reflection component and fuses the enhanced reflection component with the original image through weighting, thereby maintaining both image fidelity and the contrast of blood vessels and tissues [68]. However, this method relies on supervised training and requires a large amount of labeled data support. To solve the problem of insufficient labeled data, the application of unsupervised learning methods in image enhancement is increasing. Unsupervised deep learning methods self-learn image features to improve brightness and alleviate color distortion, providing important support for clinical decision-making [69]. Another deep unsupervised method for endoscopic image enhancement uses multi-image fusion technology to further improve image quality. This technique converts three derivative images generated from different enhancement methods into the HSI color space, then feeds the I channel image into the DerivedFuse network, which employs a novel reference-free quality metric as its loss function. The network accurately extracts and fuses features to enhance the intensity component of the original image. After enhancement, doctors can select images based on different parameter settings to suit their clinical needs [44], as shown in Figure 4. By fusing information from multiple images, this method significantly improves image detail and contrast, providing clearer visual information for doctors. The successful application of unsupervised learning highlights deep learning’s potential in addressing the challenge of limited annotated medical data, particularly when data labeling is insufficient.

Additionally, EndoL2H, a deep-learning-based super-resolution method for capsule endoscopy images, further exemplifies the importance of enhancing low-quality medical images through advanced techniques. This framework combines a conditional GAN with spatial attention to generate diagnostically relevant high-resolution images from low-resolution counterparts, ensuring the preservation of critical texture and details, even under extreme up-scaling factors. The system demonstrates excellent performance, with significant improvements in PSNR and SSIM metrics under high scaling, thereby supporting early diagnosis through enhanced visual clarity. Its application underscores the value of super-resolution in endoscopy, where limited resolution and image quality can hinder diagnostic performance [31].

**Figure 5:**
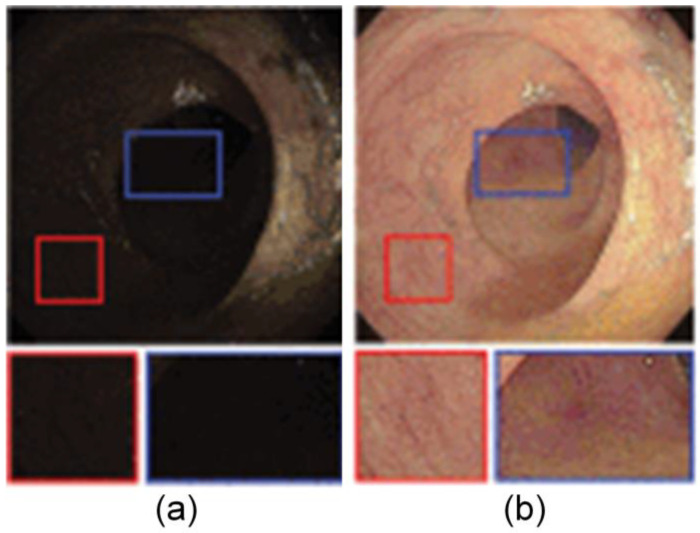
Visual results of LIE methods on the dataset. (**a**) Input image; (**b**) DPENet. In the blue and red boxes are the corresponding DOI fields (all images taken from [45]).

Additionally, deep learning models have been applied to low-light environments, such as high-speed endoscopic videos, to improve image quality in complex conditions, the improvement is significant in terms of PSNR and SSIM scores, reaching 23.79 dB and 0.88, respectively [70]. The novel deep pyramid enhancement network (DPENet), derived from deep pyramid networks (DPNs), integrates global and local features at various scales through an image pyramid framework with three parallel branches. This method has proven effective in enhancing image contrast and details under low-light conditions [45]. Visual results from the low-light image enhancement (LIE) methods applied to the dataset are shown in Figure 5. In further developments, automated frameworks have been introduced to streamline the restoration and enhancement of endoscopic images, ensuring fast processing while meeting the demands of rapid clinical diagnosis [71]. Electromagnetic interference noise removal is another key aspect of image enhancement, and researchers have developed deep learning algorithms to effectively reduce noise and enhance image clarity [72].

Finally, the application of deep learning to early gastric cancer detection has further demonstrated the practical value of image enhancement technology in clinical practice [73]. In the future, these models can be integrated into surgical navigation systems and telemedicine platforms to provide real-time and accurate diagnostic support and help doctors cope with complex lesion analysis tasks. By continuously optimizing model structures and algorithms, researchers are advancing the field of medical image analysis, broadening the future applications of deep learning in healthcare.

In summary, deep learning technology has made considerable progress in endoscopic image enhancement. By combining various methods and technologies, researchers are consistently improving image quality, diagnostic efficiency, and accuracy.

#### 3.3.4. Dynamic Tracking and Analysis

Deep learning technology has shown tremendous potential for dynamic tracking and analysis in endoscopic imaging, particularly in real-time monitoring and precise lesion localization. As deep learning algorithms continue to evolve, researchers have significantly enhanced the efficiency and accuracy of endoscopic image processing by developing innovative models, offering more reliable support for clinical diagnosis and treatment.

**Figure 6:**
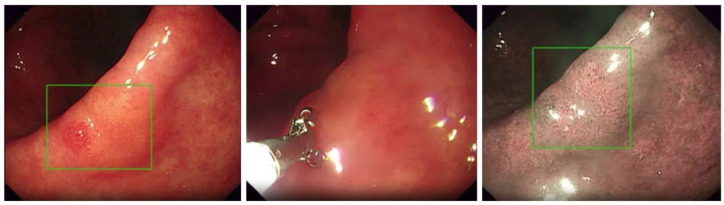
Representative examples of the clinical decision support system (CDSS). Green boxes show polyps detected by algorithm. (all images taken from [47]).

In colonoscopy, real-time endoscopic image diagnosis support systems based on deep learning have proven effective in identifying and classifying colon lesions. These systems not only enhance physicians’ work efficiency but also improve the detection rate of polyps and other lesions, highlighting the value of deep learning in dynamic endoscopic tracking [46,74]. For gastric tumor detection, a deep learning-based clinical decision support system (CDSS) was developed to automatically detect and classify tumors during real-time endoscopy, assisting in diagnosis and invasion depth prediction, with a lesion detection rate of 95.6% [47]. In the future, this system is expected to be further integrated into remote consultation and robot-assisted surgery systems to promote the popularization and development of high-quality medical services. Figure 6 presents representative identification results generated by the CDSS.

In capsule endoscopy, an automatic detection system has been developed that identifies abnormal lesions such as mucosal breaks, angioectasia, protruding lesions, and blood content in real-time [75]. By integrating Single Shot MultiBox Detector (SSD) and ResNet50, this system significantly improves the comprehensiveness of detection functions. Moreover, deep learning models have demonstrated great effectiveness in the automatic detection of early esophageal squamous cell carcinoma, with real-time monitoring and identification of abnormal esophageal lesions, thereby improving early diagnosis accuracy [76].

Regarding dynamic tracking of surgical instruments, researchers have developed deep learning-assisted robotic endoscope systems capable of tracking and locating surgical instruments in real-time, improving both the safety and precision of surgical procedures [48,77]. These advancements collectively promote the progress of endoscopic technology.

In conclusion, the application of deep learning in dynamic tracking and analysis of endoscopic images continues to evolve. By utilizing a range of deep learning models and optimized methods, the efficiency of real-time analysis has been significantly improved, offering valuable guidance for both diagnosis and surgical applications.

#### 3.3.5. Three-Dimensional Reconstruction

Deep learning technology has shown great promise in the three-dimensional (3D) reconstruction of endoscopic images. Converting two-dimensional (2D) image data into 3D models allows doctors to gain a more intuitive understanding of internal structures, thereby supporting more accurate diagnoses and treatment planning and facilitating the development of assistive tools for practitioners [78]. In recent years, significant advancements in deep learning algorithms, particularly in CNNs and multi-view stereo vision technology, have led to remarkable progress in 3D reconstruction research for endoscopic imaging.

Advancements in tracking adaptive algorithms have enhanced the accuracy of detecting and matching feature points, optimizing 3D reconstruction with the improved SuperPoint algorithm by designing a new tracking loss. This technology improves both the speed and accuracy of endoscopic 3D reconstruction, providing doctors with real-time 3D image support, which increases the number of reconstruction points and improves stability [78]. However, the robustness of the algorithm in dealing with occlusions and missing areas still needs to be further improved.

**Figure 7:**
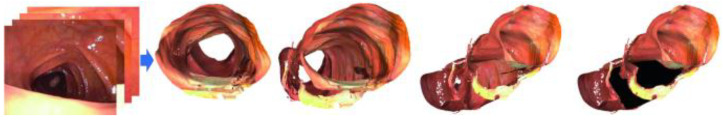
Three-dimensional reconstruction for visualization of missing colonic surface (highlighted in black in the last image, 25% surface), small colon pouches that are occluded by ridges. The missing colonic surface is highlighted in black in the last image. (all images taken from [49]).

To address this shortcoming, real-time colonoscopic 3D reconstruction technology has greatly enhanced the accuracy and efficiency of colonoscopy by dynamically reconstructing the 3D surface of the examined areas. This technology can effectively identify and fill in missing areas in the image, optimizing the doctor’s field of view and improving operational precision [49]. This is particularly advantageous when examining complex or abnormal anatomical structures, where the ability to reconstruct in 3D provides irreplaceable benefits. The results of the reconstruction are shown in Figure 7.

In summary, the application of deep learning in 3D reconstruction for endoscopic images is continuously evolving. The integration of multiple techniques has significantly improved both the accuracy and efficiency of these reconstructions. These technological advancements not only offer new perspectives for endoscopic image analysis but also provide more intuitive, real-time support for clinical applications.

## 4. Results

### 4.1. Summary of Findings

In this Results Section, we provide a comprehensive overview of the findings regarding the application of Deep Learning (DL) in enhancing endoscopic image processing. The results emphasize the positive impact of DL techniques across various applications, highlighting their effectiveness in improving diagnostic accuracy and operational efficiency. Table 3 shows the summary of findings on DL in endoscopic image processing.

### 4.2. Strengths

In this section, we highlight the significant advantages and strengths of employing DL in endoscopic image processing, underscoring its contributions to modern medical diagnostics.Table 4 shows strengths of DL in endoscopic image processing.

### 4.3. Limitations

This section outlines the challenges and constraints observed in the application of DL in endoscopic image processing. These limitations provide critical insights into areas that require further research and improvement. Table 5 shows limitations of DL in endoscopic image processing.

## 5. Discussion

The application of deep learning to endoscopic image processing represents a transformative advancement in medical diagnostics. Techniques based on convolutional neural networks have significantly improved the resolution, clarity, and diagnostic accuracy of endoscopic images. Through feature extraction, pattern recognition, and automated segmentation, deep learning models are surpassing the limitations of traditional image processing methods, offering faster and more reliable insights for clinical use.

Deep learning models, such as CNNs, excel at interpreting complex visual data, automatically identifying subtle patterns that may elude human perception. This capability is especially valuable for detecting early-stage malignancies or subtle abnormalities that could otherwise go unnoticed. The automation of detection and classification processes minimizes the risk of human error, leading to more accurate diagnoses and improved clinical outcomes. Furthermore, methods like EndoL2H leverage super-resolution techniques to enhance image quality, ensuring that even low-resolution endoscopic images retain diagnostically critical details [31]. Similarly, multi-image fusion approaches, as described by Huang et al. (2022), improve image brightness and color accuracy through unsupervised learning techniques, supporting enhanced visualization in clinical settings [64].

Despite these advancements, several challenges hinder the widespread adoption of deep learning in clinical practice. A major concern is the computational demand required to train and operate these models. Training deep networks requires substantial processing power and large-scale datasets, which may not always be available in medical institutions. This challenge is particularly relevant in healthcare settings where data scarcity is common, making it difficult to train models effectively using supervised learning approaches. As Hamid (2023) points out, data-centric AI approaches, which emphasize optimizing datasets alongside models, are critical for addressing these limitations [79].

Another challenge lies in ensuring that deep learning models maintain robustness across different clinical environments. Variability in endoscopic equipment, patient demographics, and imaging protocols can lead to domain shifts, which may negatively affect model performance. Developing models that are generalizable and capable of maintaining accuracy across diverse datasets remains a priority. Data-centric solutions, as emphasized by Hamid and Braun (2019), suggest that focusing on creating adaptable, high-quality datasets is essential for improving the scalability of AI models in healthcare [80].

Additionally, the “black box” nature of many deep learning models poses interpretability challenges. In medical diagnostics, transparency and accountability are essential, as clinicians need to understand the rationale behind AI-generated outputs to make informed decisions. While interpretability-focused models, such as those developed by Mukhtorov et al. (2023), provide some solutions, further research is needed to balance model complexity with explainability [61].

Despite these challenges, the potential of deep learning to alleviate the workload of clinicians is immense. Automating routine tasks allows healthcare providers to focus more on patient care, ultimately improving the efficiency of healthcare delivery. By integrating deep learning models into diagnostic workflows, clinicians can benefit from faster and more precise diagnoses, leading to better patient outcomes. As advancements continue, the field will need to prioritize both computational efficiency and transparency to ensure that deep learning tools are seamlessly integrated into clinical practice.

In conclusion, while the integration of deep learning into endoscopic image analysis holds great promise, addressing the challenges of computational demands, data scarcity, and interpretability will be crucial for its success. By adopting strategies that balance data-centric and model-centric approaches, researchers can develop more efficient and adaptable models that meet the demands of modern healthcare systems [79,80].

## 6. Conclusions

This paper provides a comprehensive review of deep learning applications in endoscopic image processing, covering advancements in image enhancement, segmentation, classification, dynamic tracking, and 3D reconstruction. Key contributions include emphasizing the impact of super-resolution and multi-image fusion techniques, exploring unsupervised learning to address data scarcity, and highlighting model scalability across diverse clinical settings.

The analysis identifies future directions, including developing lightweight, interpretable models for efficient clinical use and exploring data-efficient techniques like domain adaptation to address limited annotated datasets. By aligning emerging AI trends with clinical needs, this work offers a roadmap for future research, aiming to enhance diagnostic accuracy, improve patient care, and drive the integration of deep learning into routine medical practice.

## Figures and Tables

**Table 1:** Comparative overview of review papers.

Review Paper	Focus	Strengths	Gaps	How This Review Fills the Gap
Shen et al. (2017) [7]	Deep learning in medical imaging	Provides a comprehensive overview of deep learning models	Insufficient focus on real-time endoscopic applications	Addresses endoscopy-specific challenges like real-time processing
Alagappan et al. (2018) [10]	Future trends in AI for endoscopy	Predicts future AI impacts	Limited focus on current optimization strategies	Bridges research advancements with clinical implementation
Choi et al. (2020) [8]	CNN-based endoscopic imaging	Highlights polyp detection and technical evolution	Limited scope to segmentation and polyp detection	Addresses advanced optimizations like noise reduction
Pannala et al. (2020) [9]	Practical AI in endoscopy	Emphasizes real-time CADe systems	Lacks discussion on super-resolution and denoising	Incorporates advanced image enhancement strategies
Zhuang et al. (2023) [12]	Digestive system image processing	Focused review of deep learning in digestive imaging	Limited cross-disciplinary scope	We integrate insights across broader endoscopic applications

**Table 2:** Overview of Applications and Popular Models.

Application	Popular Models	Key Advances
Image segmentation	U-Net SegNet	Early cancer detection [38] Polyp segmentation [39] Bleeding area monitoring [40]
Image Classification	ResNet-50 FCN	GERD classification [41] Nasopharyngeal malignancy [42] Early gastric cancer [43]
Image Enhancement	CNNs EndoL2H	Super-resolution [31] Multi-image fusion [44] Low-light enhancement [45]
Dynamic Tracking and Analysis	SegNet	Polyp detection [46] Tumor classification [47] Surgical instrument tracking [48]
3D Reconstruction	SuperPoint Multi-view	Colonoscopy reconstruction [49] Real-time 3D surface filling Tracking adaptive algorithms

**Table 3:** Summary of findings on DL in endoscopic image processing.

Findings	Description
Application Areas	DL is utilized in various areas, including classification, reconstruction, image enhancement, segmentation, and real-time pathology recognition across different types of endoscopic procedures, such as gastrointestinal and respiratory endoscopy.
Performance Metrics	Studies have shown that DL algorithms can enhance accuracy rates in lesion detection, particularly in identifying precancerous lesions and small tumors.
Impact on Workflow	DL systems have reduced the processing time for real-time analysis, allowing clinicians to make faster and more informed decisions during procedures.
Comparison with Traditional Methods	DL methods generally outperform traditional algorithms, such as manual review and conventional image processing techniques, in terms of speed and accuracy, particularly in polyp detection.
Quality of Image Output	Enhanced image quality through techniques like super-resolution has resulted in clearer visualization of details, aiding in more accurate diagnoses.

**Table 4:** Strengths of DL in endoscopic image processing.

Strengths	Description
Enhanced Diagnostic Accuracy	DL techniques have been shown to significantly improve diagnostic accuracy, particularly in detecting early-stage malignancies and subtle anatomical abnormalities that might be overlooked by human observers.
Automation of Image Analysis	By automating repetitive tasks like image segmentation and classification, DL reduces the workload for clinicians, allowing them to focus on patient care and complex decision-making.
Real-time Processing Capabilities	DL systems facilitate real-time analysis, which is critical during endoscopic procedures, enabling immediate feedback and decision support to clinicians.
Adaptability and Continuous Learning	Many DL models can adapt to new data inputs and learn from ongoing clinical experiences, improving their accuracy and reliability over time.
Potential for Personalized Medicine	With ongoing advancements, DL technologies hold the promise of enabling personalized medicine by tailoring diagnostic and therapeutic approaches to individual patient profiles.

**Table 5:** Limitations of DL in endoscopic image processing.

Limitations	Description
Data Dependency	A major limitation is the high dependency on large, annotated datasets for training DL models. Many institutions face challenges in obtaining sufficiently large and diverse datasets, which may lead to biased model performance.
Generalization Issues	DL models may struggle to generalize across different endoscopic modalities and variations in imaging protocols, which can affect diagnostic accuracy when applied to new settings.
Interpretability	The “black box” nature of DL algorithms presents challenges in clinical settings, as clinicians often find it difficult to interpret the rationale behind AI-generated outputs, which is crucial for making informed medical decisions.
Computational Requirements	The computational resources needed for training and implementing DL models are substantial, often requiring advanced hardware that may not be readily available in all healthcare settings.

## Data Availability

This is a review of published articles, and all the articles are publicly available.
